# Impact of COVID-19 on international medical education and the future plans of medical students in Japan

**DOI:** 10.12688/mep.18953.2

**Published:** 2022-06-16

**Authors:** Houman Goudarzi, Masahiro Onozawa, Makoto Takahashi

**Affiliations:** 1 Center for Medical Education and International Relations, Faculty of Medicine and Graduate School of Medicine, Hokkaido University,, Sapporo, Japan, 060-8638, Japan; 2 Clinical Training Center, Hokkaido University Hospital, Sapporo, Hokkaido, 060-8638, Japan

**Keywords:** COVID-19 pandemic, medical education, medical students, future plans, outbound student mobility

## Abstract

**Background**: The aim of this study was to assess the impact of the coronavirus disease 2019 (COVID-19) pandemic on the current study methods and future plans of medical students compared to those in the pre-pandemic period.

**Methods:** Second-grade medical students reported their academic experiences, study methods, and future career plans before (between 2016 and 2019) and during the pandemic (2020) using a questionnaire-based survey at Hokkaido University, Japan (n = 534).

**Results:** From 2016 to 2019, we found an increasing trend for participation in short-term international exchange programs, taking the United States Medical Licensing Examination (USMLE), clinical training, and undertaking research abroad among the students. However, these percentages significantly declined (to 35.5%) during the COVID-19 pandemic in 2020 for all the assessed future plans, including short-term exchange programs (-27.9%), taking USMLE (-19.8%), clinical training (-24.5%), and undertaking research abroad (-13.2%) compared to 2019, wherein 67.9% of the students wished to have at least one of these four above-mentioned academic activities.

**Conclusions:** The COVID-19 pandemic adversely and significantly influenced our medical students’ plans to go abroad for clinical and research training. Future studies are warranted to assess the long-term influence of this pandemic on the career planning of medical students.

## Introduction

The internationalization of higher education is a relatively new phenomenon; however, the concept of internationalization is broad and varied. Over the last 30 years, several research and education programs in different countries have been the motor for a broader and more strategic approach to internationalization in higher education, which is defined as “the intentional process of integrating an international, intercultural, or global dimension into the purpose, functions, and delivery of post-secondary education to enhance the quality of education and research for all students and staff and to make a meaningful contribution to society” ( De Wit *et al*., 2015). In the 21st century, with extraordinary rapid changes, reforming professional education is inevitable. It is necessary to adapt education to strengthen health systems based on transnational, multi-professional, and long-term perspectives to respond needs of humans ( Frenk *et al*., 2010).

The most important benefits of international higher education are increased international awareness of the deeper engagement with global issues by students and improved quality of teaching and learning in Asia and the Pacific ( De Wit *et al*., 2015; Stevens & Simmonds Goulbourne, 2012). A recent study examined the US clinical experience for Japanese international medical graduates and benefits of US training (Heist *et al.*, 2020). Although medical education and the overall medical systems in the U.S. and Japan are different, international exposure of young physicians and direct learning in the two disparate systems will bring about the integration and the improvement of the medical systems in both countries. At a time of global interconnectedness, the internationalization of medical education has become an important part of medical education. Although several countries, including Japan, had implemented various strategies recently to enhance inbound and outbound student mobility, the coronavirus disease 2019 (COVID-19) pandemic halted international travel.

The World Health Organization (WHO) upgraded the COVID-19 outbreak to pandemic status on March 11, 2020. This has led to serious implications in almost all aspects of human life. The pandemic has also had a huge impact on the educational system, and in particular, medical education to a significant extent ( Cleland *et al*., 2020). Medical schools have experienced dramatic disruptions in every aspect of medical education. Universities shifted the classes to online lessons and asked students to stay at home. In-person pre-clinical educational sessions were shifted to remote learning experiences, and student participation in all direct patient contact activities were paused or limited. Most education-related travels, including exchange student programs, electives, and international examinations, such as the United States Medical Licensing Examination (USMLE), have been canceled, suspended, or modified.

Although Japan has been promoting teaching medicine in English in recent decades by implementing medical English courses in the curriculum in pre-clerkship and during clerkship for medical students, medicine in Japan is mostly taught in Japanese. Medical students in Japan tend to study without ever opening English-written textbooks. Consequently, English proficiency has stagnated, and the national results of English proficiency tests, such as Test of English as a Foreign Language (TOEFL) and International English Language Testing System (IELTS), are generally lower than those of many other advanced countries ( EF EPI, 2018; IELTS Test taker performance, 2019). In addition, national board exams for medical doctors have been performed in Japanese, although a few English questions have been introduced in recent years. Therefore, for some students, it is difficult to maintain their motivation to learn English for medical purposes and engage in international medical education and activities in Japan. The motivation to learn English significantly depends on their interest in global career development ( Onozawa *et al*., 2020).

We conducted a medical English course for second-grade medical students, including a 15-session course starting in April every year. Our course is a bilingual (Japanese and English) inspiring course that focuses on the core competencies of English for medical purposes, including doctor–patient (taking history and physical examination) and doctor–doctor (evidence-based medicine, medical terminology, scientific presentation, etc.) communication. Since 2016, we have been conducting a questionnaire-based survey of our students before starting our course for a detailed characterization of second-grade medical students in the spring semester at Hokkaido University. To address the potential impact of the COVID-19 pandemic on academic achievements/plans of our students, in this study, we aimed to examine the potential impact of the COVID-19 pandemic on the academic-related outcomes of our students, such as current study methods and future plans by comparing the pre-pandemic (2016–2019) and pandemic (2020) periods.

## Methods

### Study design

This study was a cross-sectional study between April 2016 and April 2020 at Hokkaido University, Sapporo, Japan. All second-grade medical students were eligible to complete participate in the current study. Using a questionnaire-based survey, the students at the first class in our course reported their academic experiences at the beginning of the course from 2016 to 2020. Of 541 eligible students, 7 students did not agree to participate in the current study. Ultimately, 534 took participation in our study (response rate: 98.7%) as follows: 108 in 2016, 108 in 2017, 108 in 2018, 106 in 2019, and 104 in 2020 ( Table 1).

**Table 1.** Characteristics of the study participants (n = 534).

Year	Number	Female, n (%)	National Center Test for University Admissions (English test score) *	Students passed standard English exams, n (%) ^+^
2016	108	24 (22.2)	184.1 ± 10.2	52 (48.1)
2017	108	24 (22.2)	185.3 ±10.5	39 (36.1)
2018	108	21 (19.4)	191.2 ± 6.7	44 (40.7)
2019	106	19 (17.9)	183.7 ± 10.5	47 (44.3)
2020	104	18 (17.3)	183.3 ± 6.5	45 (43.2)

* Average ± standard deviation, out of 200 ^+^ IELTS (International English Language Testing System), TOEIC (Test of English for International Communication), TOFLE (Test of English as a Foreign Language), etc.

### Questionnaire

A 15-item questionnaire was designed by one of the authors (M.O.) ( Onozawa *et al*., 2020) and discussed for content and construct validity (H.G., M.T.). No major changes were made to the preliminary version of the questionnaire. The questionnaire included closed- and open-ended questions, relating to the following themes:

(1) How have the students been exposed to and learned English (English experience), including participation in English conversation school, private lessons, volunteer activity, living in a foreign country, traveling abroad, short homestay abroad?
(2) How are the students studying English (current study methods) using radio, TV, podcasts, watching online movies, medical textbooks, journals, newspapers, conversation schools, private lessons, traveling abroad, international exchange student programs, and English skill examinations?
(3) What type of international academic activities do the students wish in their future (wish list), such as international exchange student programs, taking the USMLE, clinical training abroad, doing research abroad?

From 2016 to 2019, we distributed paper-based questionnaires and collected data on the above-mentioned questions. However, the survey was conducted on an online platform in 2020 due to the COVID-19 pandemic and online education. For all the three main categories of the above-mentioned questions, the students could select more than one option. The questionnaire can be found as *Extended data* ( Goudarzi *et al.,* 2022b).

### Ethical considerations

After explanation of the aim of the current study, we asked all second grade medical students in our school to participate in the present study at the first lesson of our medical English course. All participants gave written informed consent. No financial or other incentives were provided for participation. The questionnaire had an opt-out item for students who did not want to be included in the data analysis. Ethical approval for this study was obtained from the Institutional Review Board of the Faculty of Medicine and Graduate School of Medicine, Hokkaido University (20–040).

### Statistical analysis

Descriptive data were reported as percentages or means with standard deviation. Statistical analyses were performed using the statistical software package JMP version 14 (SAS Institute Inc., Cary, NC).

## Results

**Figure 1.**
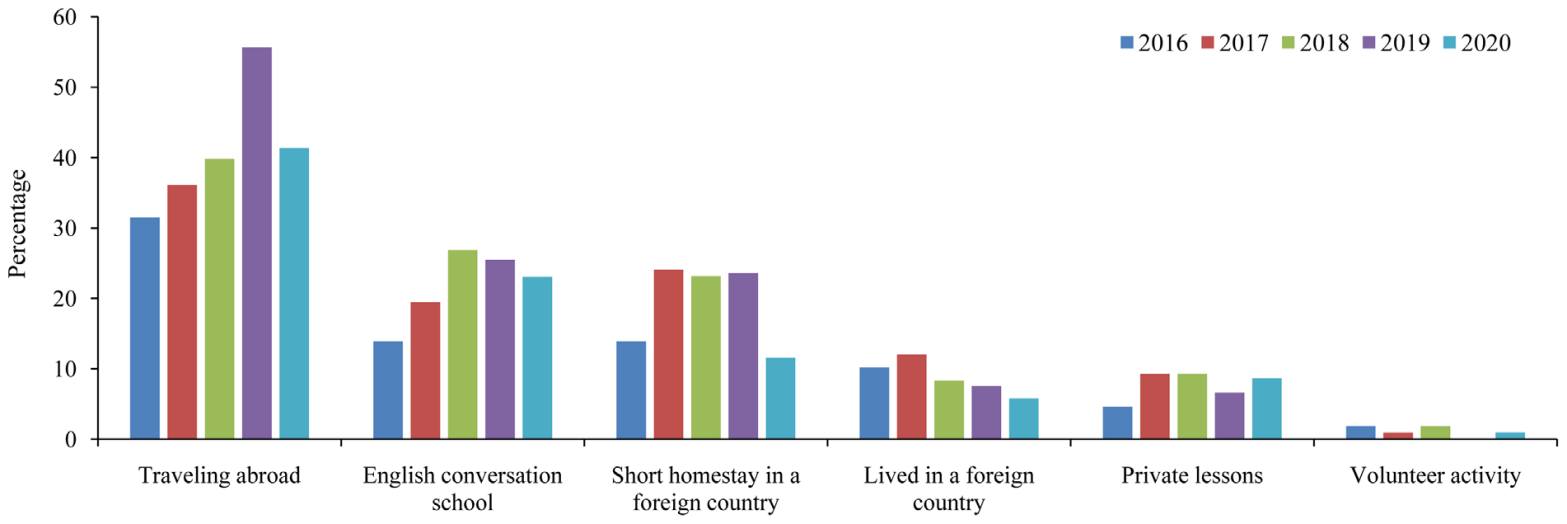
Previous English experience (exposure) in Japanese medical students between 2016 and 2020.

After excluding students who did not provide consent forms (n = 7), we collected data from 534 students over five years of the current study period from 2016 to 2020: 108 in 2016, 108 in 2017, 108 in 2018, 106 in 2019, and 104 in 2020 ( Table 1) ( Goudarzi *et al.,* 2022a). Of the students, 19.8% were female. Figure 1 shows the previous English experience (exposure) of students from 2016 to 2020. Traveling abroad was the most common experience, followed by a short homestay in a foreign country and participation in a conversation school. However, volunteer activities were uncommon among our students. We did not observe an evident trend for the assessed items, except for a declining trend for living abroad among students from 2016 to 2020.

**Figure 2.**
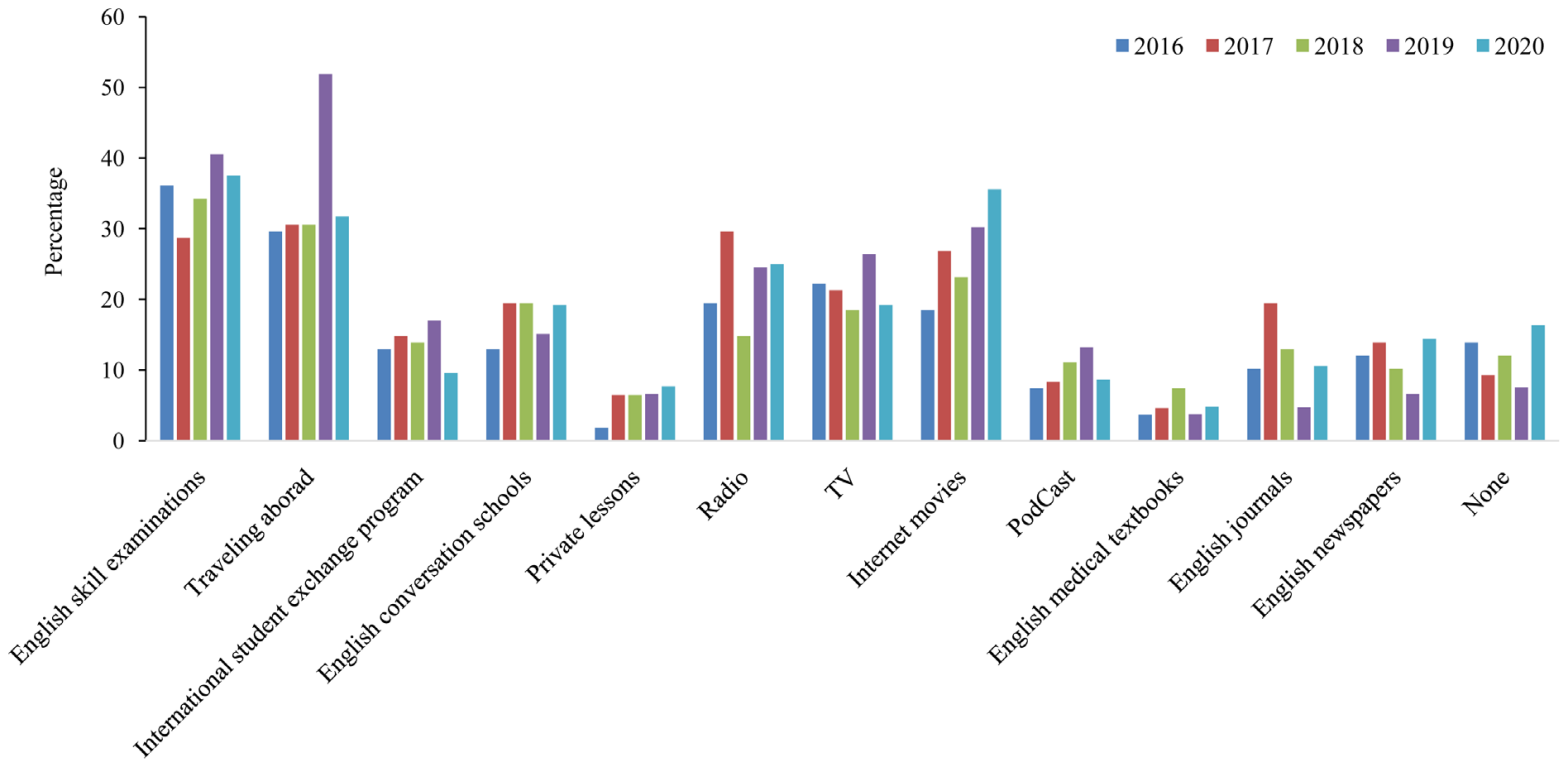
Current English study methods of Japanese medical students between 2016 and 2020.

As shown in Figure 2, an evaluation of current study methods revealed that apart from traveling abroad and English skill examinations, watching online movies was the most popular study method among our students. Conversely, reading medical textbooks in English was the least popular method. Although we did not find a monotonous trend for the current English learning methods, watching online movies showed an increasing trend among students with the highest percentage during the COVID-19 pandemic. Notably, approximately 12% (ranging from 7.5% in 2019 to 16.3% in 2020) of the students did not study English at the time of conducting the survey.

**Figure 3.**
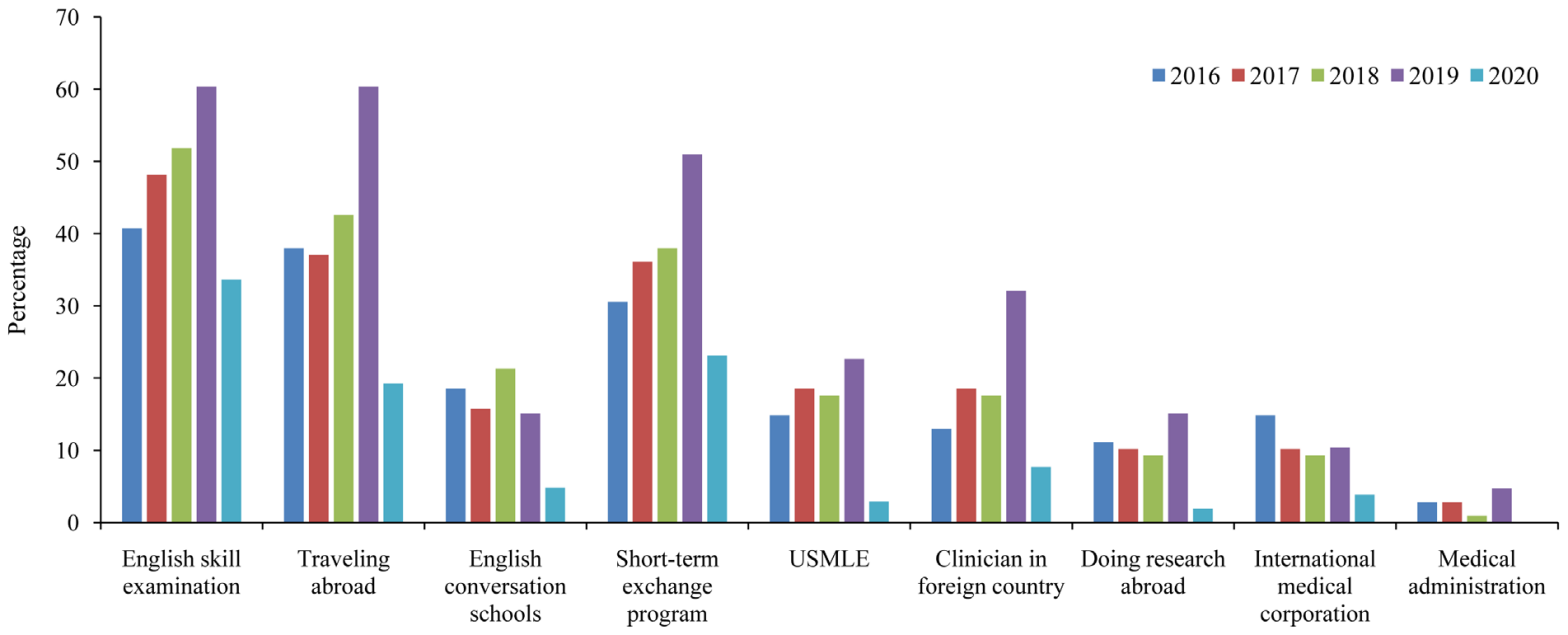
Wish list of Japanese medical students between 2016 and 2020. USMLE=United States Medical Licensing Examination.

From 2016 to 2019 (before the COVID-19 pandemic), we found an increasing trend for taking English skill examinations, such as IELTS, TOEFL, Test of English for International Communication (TOEIC) ( Figure 3). In addition, participation in short-term international exchange programs, taking the USMLE, clinical training, and conducting research abroad showed an increasing trend in the pre-pandemic era. In 2019, the percentages of students who planned for academic-related programs were 50.9% for the short-term exchange program, 22.6% for taking the USMLE, 32.1% for clinical training, and 15.1% for doing research abroad. In 2019, 67.9% of the students wished to have at least one of the four above-mentioned academic activities, which indicated the willingness of our students to get involved in various international programs prior to the COVID-19 pandemic. However, these percentages significantly declined owing to the COVID-19 pandemic in 2020 for all assessed future plans, including short-term exchange programs (23.0%), taking the USMLE (2.9%), clinical training (7.7%), and undertaking research abroad (1.9%). In total, only 35.5% of our students wished to engage in such activities in 2020.

## Discussion

To the best of our knowledge, this is the first report on the outbound mobility of medical students during the COVID-19 pandemic. We found promisingly increasing trends in international academic plans and activities from 2016 to 2019. About 68% of our students planned to engage in at least one of the academic-related activities; however, this number declined to 35% during the COVID-19 pandemic in 2020. Therefore, we observed a profound adverse effect of the COVID-19 pandemic on the trend of future academic planning among medical students in the pre-clerkship period, with a significant decline in studying or training abroad and intention to take international medical examinations. Notably, COVID-19 may affect the future career pathways of physicians, and such potential enduring transformation should be monitored in future longitudinal studies regarding the permanent change in medical education, academic achievements, and the careers of medical students and physicians.

We found an increasing trend regarding studying abroad, conducting research, or clinical training abroad among Japanese medical students between 2016 and 2019 before the COVID-19 pandemic. This could be a consequence of recent government and higher education institutions' attention turned toward outward student mobility in Japan ( Bradford, 2015). Japan experienced a significant decline of Japanese students going abroad after its peak in the first half of the 2000s, and this was called “inward-looking,” which implied that many young Japanese hesitated about going abroad for their studies. Therefore, in the recent decade, the Ministry of Education, Culture, Sports, Science, and Technology and other government bodies commenced the implementation of initiatives designed to internationalize the education system in Japan. In 2012, the Japanese government increased funding for outbound student mobility, and several programs, including collaborative mobility programs, joint degrees, credit transfer systems, and scholarships, were implemented. In addition, the Japanese government launched the Japan Revitalization Strategy in 2013 to double the number of students studying abroad by 2020 ( Bradford, 2015; Kuroda *et al*., 2018). Furthermore, recent studies reported that the number of university exchange agreements increased significantly, especially for students who wished to study abroad for a short period of time ( Horio, 2017; Kuroda *et al*., 2018). At least, partly, the increasing trend of outbound interest of the students between 2016 and 2019 in our study could be explained by government programs and university agreements, and the current pandemic represents a significant disruption in such established programs.

Across the five years of the study, around 16% of students who participated in 2020, reported they did not study English, which was the highest compared to the other years ( Figure 2, 16.3% vs. 7.5% in 2019). It is a very concerning result which could be, at least partially, because of the loss of motivation of the students during the COVID-19 era. Further studies are necessary to demonstrate underlying factors that result in the unwillingness of medical students to learn foreign languages, including demotivation (Riasati *et al*., 2018). Such demotivation due to the pandemic may have consequent language learning failure for medical students ( Vakilifard *et al*., 2020). A recent German study reported that suitable digital learning formats and social support are important study resources for engagement during the COVID-19 pandemic among health and social professions students ( Koob *et al*., 2021). In addition, universities and health institutions should provide beneficial teaching formats and innovative methods to support students during major life events.

On the other hand, watching online movies was the most popular English study method in 2020, almost double of that in 2016 (35.6% vs. 18.5%). This might reflect the recent expanding availability of subscription video-on-demand services, which are preferentially chosen in the stay-home policy during the COVID-19 era. It would be better to conduct more surveys and face-to-face interviews to clarify the content of popular online learning programs among medical students and apply promising methods in future studies to enhance online learning strategies.

We found a remarkable influence of the COVID-19 pandemic on the future plans of our students, mainly regarding student mobility ( Figure 3). A recent report in mainland China and Hong Kong, mainly from undergraduate schools (n=2,739), showed that 84% of students reported no interest in studying abroad even after the COVID-19 pandemic ( Mok *et al*., 2021); although they examined under- and post-graduate students, rather than medical students, their data were in line with our results. They also found that the students who continue to pursue further study abroad think about Asian countries, such as Hong Kong, Japan, and Taiwan, in addition to the UK and the US, thereby suggesting a shifting mobility flow of international students. Another recent study reported that the most important concerns of international graduate students during the pandemic in the US were maintaining good health during their studies (67%) and understanding US medical insurance and obtaining health services (53%) ( Chirikov & Soria, 2020). Therefore, medical students in Japan and Asian countries may think about countries having a better control of the pandemic as an ideal destination for international higher education in the future. In addition, we believe that the current pandemic has to influence different aspects of education in the world, not limited to medical education. Therefore, such results could be observed among medicine-related post-graduates and researchers.

Several international organizations, such as UNESCO, World Education Services, and the British Council, recently predicted a decline in student mobility to major destination countries, and our results confirmed this ( Durnin, 2020; Goris, 2020; Schulmann, 2020). The experience of participating in international clinical rotations and residency provides a wide range of advantages from both practical and academic standpoints. In addition to broadening medical knowledge, such opportunities enable passionate candidates to explore the subject in-depth and gain real-world experience that they would not be able to obtain at their medical schools. It may also encourage the young generation of physicians to practice medicine among underserved and multicultural populations, which are always concerned with health systems, especially in developed countries. However, the US and European countries may have been influenced by the shortage of residents and medical doctors from foreign countries during the pandemic. Approximately 250,000 foreign-born doctors are practicing in the US, thus playing a key role in providing health care for millions of Americans ( American Immigration Council, 2021). Moreover, the UK, compared to other European countries, has more foreign doctors who play a vital role in running Britain’s publicly funded health service. Although most Japanese residents/doctors return to Japan after completing their training in a foreign country, the declining motivation during the COVID-19 pandemic and beyond to participate in international clerkship and residency in Asian countries will have serious consequences in the future for health care systems on a global scale.

Globalized medical education needs medical students to understand the global burden of diseases and epidemiology as well as disparities and inequities in global health systems. Consequently, all medical schools should incorporate programs for global health training and support inbound and outbound medical students’ mobility. In the US, medical school graduates join residency programs with international health experiences ( Drain *et al*., 2007). Although we did not find such a survey in Japan, this number seems to be significantly lower than that in the US. Additionally, our current study suggests that this number may decline during the COVID-19 pandemic and beyond. Therefore, placing more emphasis on global epidemiology courses and even the development of Master of Public Health programs in addition to other innovations in medical education would be beneficial toward training the next generation of physicians oriented to potential future pandemics and be able to contribute to the improvement of human health globally ( Gibbs, 2020; Sangha & Hao, 2021).

The WHO declared the COVID-19 outbreak a global pandemic on March 11, 2020 (4). In late March, the Japanese Ministry of Health, Labor, and Welfare announced the daily increasing number of confirmed cases, and the Japan Medical Association asked the government to declare a state of emergency. A couple of days before our survey in mid-April, 2020, the Prime Minister declared the first nationwide state of emergency. Therefore, the timing of the survey could have significantly influenced students’ minds and the results of the current study, while the world faced an unprecedented shock in addition to the unavailability of vaccines at that moment. However, because of the potential negative impact of declined outbound medical student mobility during the COVID-19 pandemic and beyond on the drive to nurture the next generation of physicians, it is necessary to follow up with medical students with longitudinal surveys and create supportive systems to develop a meaningful response, overcome barriers, and propose solutions to repair the damage caused by COVID-19’s interruptions to learning trajectories.

Although the pandemic stressed the health care systems and medical education, it could also be a catalyst for the transformation of medical education (Lucey *et al.*, 2020). The tremendous stress of the pandemic on education and healthcare systems may provide an opportunity to reassess the entire medical education, healthcare preparedness, and delivery. COVID-19 has been pushing universities to change their attitudes toward the curricula, methods of teaching, learning, and assessment. Hopefully, the COVID-19 pandemic will force education to a higher level and help the preparedness of universities for pandemics or a global health crisis in the future.

The current study has some limitations. We collected data from students between 2016 and 2019 via paper-based questionnaires. However, because of the prevalence of remote education in 2020, we collected such information using online questionnaires. This could influence the results; however, based on the adequate orientation of the students before completing the survey and monitoring of the students during the course, comparing the results with the survey conducted at the end of the course suggested a minimal possibility of such bias. Additionally, we looked at the characteristics of the medical students each year between 2016 and 2020 to find any possible differences across the years that influence the results of the current study. However, we found a comparable sex ratio, university entrance exam scores, and percentage of students who passed standard English proficiency tests, such as IELTS, TOEFL, and TOEIC, each year. Furthermore, we assessed the English language proficiency of the students, including reading, writing, listening, and speaking skills, using a self-administered questionnaire and found rather similar proficiency levels among students each year, indicating a low possibility of the influence of students’ English ability on the obtained results. We conducted this survey before clinical clerkship, and attitudes toward future career paths may change for the second-grade medical students over time. Therefore, it is necessary to follow up with the students to examine whether intent translates into action after graduation. Our survey is based on a single medical school in Japan. Therefore, medical students' attitudes in our university may not be extrapolated to all national and private schools in Japan. Further multi-institutional surveys are necessary to replicate our results. In addition, we did not specifically examine the underlying cause(s) for a significant decline in interest in studying or working abroad among the students' in 2020. Therefore further quantitative research is needed to underpin the underlying reasons in detail.

Some countries are planning to facilitate international travel with vaccination passports; however, much uncertainty persists for re-opening global medical communication, such as student and residency programs. For future research, more comprehensive studies on medical education, not merely a wish list of medical students, with longitudinal surveys are warranted to clarify the adverse effects of the COVID-19 pandemic on medical students and medical staff. Such studies will help to address the difficulties students face regarding their future goals and careers. In addition, close monitoring of education-related outcomes may facilitate the implementation of relevant education-oriented strategies, updating curricula, and supporting students to nurture the next generation of brilliant and motivated clinicians, physician-scientists, and researchers.

## Conclusion

Although we observed an increasing trend for student outbound mobility among our medical students between 2016 and 2019, the COVID-19 pandemic adversely influenced this trend in 2020. Further longitudinal studies are necessary to determine the long-term effects of the pandemic on the trajectory of medical education and student mobility.

## Take home messages

- Before the COVID-19 pandemic, we found an increasing trend for participation in short-term international exchange programs, taking the USMLE, clinical training, and undertaking research abroad among our medical students between 2016 and 2019.
- In 2020, we observed a significant decline in students’ wish for outbound mobility, including short-term exchange programs (-27.9%), taking the USMLE (-19.8%), clinical training (-24.5%), and undergoing research abroad (-12.3%) compared to 2019.
- In 2019, 67.9% of the students wished to engage in at least one of these four above-mentioned academic activities; however, it declined to 35.5% in 2020 during the pandemic.
- The COVID-19 pandemic adversely and significantly influenced our medical students’ plans to go abroad for clinical and research training.
- Further longitudinal studies are necessary to determine the long-term effects of the pandemic on the trajectory of medical education and student mobility.

## Data availability

### Underlying data

DRYAD: Impact of COVID-19 on international medical education. https://doi.org/10.5061/dryad.0vt4b8h1b ( Goudarzi *et al.,* 2022a).

This project contains the following underlying data:

- Impact_of_COVID-19_on_international_medical_education.xlsx
- README_(Impact_of_COVID-19_on_international_medical__education).xlsx

Data are available under the terms of the Creative Commons Zero "No rights reserved" data waiver (CC0 1.0 Public domain dedication).

### Extended data

Zenodo: Impact of COVID-19 on international medical education. https://doi.org/10.5281/zenodo.6069426 Goudarzi *et al.,* 2022b.

This project contains the following extended data:

- Questionnaire_2022.02.09.docx

Data are available under the terms of the Creative Commons Attribution 4.0 International license (CC-BY 4.0).
