# Supplementary material for: Impact of COVID-19 on international medical education and the future plans of medical students in Japan
Source: MedEdPublish (2016). 2022 Jun 16;12:15. Originally published 2022 Mar 3. [Version 2] doi: 10.12688/mep.18953.2 (PMC9370090; doi:10.12688/mep.18953.2)
Supplement: Peer review history

# Peer review: Reviewer response for version 2

**DOI:** 10.21956/mep.20563.r32148

**Published:** 2022-06-24

**Authors:** Jay Yang, MD, PhD, MSEE

**Affiliations:** 1 Emeritus Professor, University of Wisconsin, Madison, WI, USA

The authors have addressed most of my previous concerns and I have no further comments.

Have any limitations of the research been acknowledged?

Partly

Is the work clearly and accurately presented and does it cite the current literature?

Yes

If applicable, is the statistical analysis and its interpretation appropriate?

No

Are all the source data underlying the results available to ensure full reproducibility?

Yes

Is the study design appropriate and is the work technically sound?

Yes

Are the conclusions drawn adequately supported by the results?

Yes

Are sufficient details of methods and analysis provided to allow replication by others?

Yes

Reviewer Expertise:

Cellular and molecular medicine, clinical anesthesiology, medical education

I confirm that I have read this submission and believe that I have an appropriate level of expertise to confirm that it is of an acceptable scientific standard.

# Peer review: Reviewer response for version 1

**DOI:** 10.21956/mep.20310.r31822

**Published:** 2022-04-28

**Authors:** Forman Erwin Siagian

**Affiliations:** 1 Department of Parasitology, School of Medicine, Universitas Kristen Indonesia, Jakarta, Indonesia

Firstly, I would like to express my compliment to these authors due to their effort to examine the impact of covid on global medical education and combine it with the plans/futures of Japanese medical students.

 This research positively reveals how higher education, especially medicine, has responded to the pandemic for its survival. In my opinion, the limitations of the research have been acknowledged partly, due to probably because of the qualitative approach to writing and this does not become an obstacle for the whole article in contributing positively to the field of medical science.

 The reason for my statement “the limitations of the research have been acknowledged partly, due to probably due to the qualitative approach to writing” is as follows:

  

1. In the conclusion section, the author states that covid reduces the interest of medical students to go study abroad. The underlying reason for this is not stated clearly, I think this is probably because the possible answer is not covered in the questionnaires distributed. So this is a research limitation that has not been revealed in this study yet and further quantitative research is needed.
  I quote Queirós (2017) ^1^, who states “ Qualitative research is therefore concerned with aspects of reality that cannot be quantified, focusing on the understanding and explanation of the dynamics of social relations. Qualitative research works with the universe of meanings, motives, aspirations, beliefs, values, and attitudes, which corresponds to a deeper space of relationships, processes, and phenomena that cannot be reduced to the operationalization of variables.”
  Scientific research adopts qualitative and quantitative methodologies in the modeling and analysis of numerous phenomena. The qualitative methodology intends to understand a complex reality and the meaning of actions in a given context. On the other hand, the quantitative methodology seeks to obtain accurate and reliable measurements that allow statistical analysis. Both methodologies offer a set of methods, potentialities, and limitations that must be explored.
2. In the discussion section, the author states that “It is a very concerning result which could be, at least partially, because of the loss of motivation of the students during the COVID-19 era.” It would be very interesting to carry out further research to find out whether it is the loss of motivation that underlies the unwillingness of medical students to learn foreign languages or perhaps any other reason. ^2^^ ^Demotivation has negative effects on language learning processes and outcomes. ^3^ Actually, many factors influence the study engagement of health and social professions students during the COVID-19 pandemic. ^4^

That's my opinion regarding the limitations of this research, but it should be understood that my personal opinion aims to make this article better.

Have any limitations of the research been acknowledged?

Partly

Is the work clearly and accurately presented and does it cite the current literature?

Yes

If applicable, is the statistical analysis and its interpretation appropriate?

Yes

Are all the source data underlying the results available to ensure full reproducibility?

Yes

Is the study design appropriate and is the work technically sound?

Yes

Are the conclusions drawn adequately supported by the results?

Yes

Are sufficient details of methods and analysis provided to allow replication by others?

Yes

Reviewer Expertise:

Parasitology, Medical Education, questionnaire-based survey research, human behaviour

I confirm that I have read this submission and believe that I have an appropriate level of expertise to confirm that it is of an acceptable scientific standard.

## References

[1] : STRENGTHS AND LIMITATIONS OF QUALITATIVE AND QUANTITATIVE RESEARCH METHODS. *European Journal of Education Studies* .2017;9(3) : 10.5281/zenodo.887089 10.5281/zenodo.887089

[2] : Willingness to speak English among foreign language learners: A causal model. *Cogent Education* .2018;5(1) : 10.1080/2331186X.2018.1455332 10.1080/2331186X.2018.1455332

[3] : Investigating demotivating factors in foreign language learners: The case of non-Iranian Persian language learners. *Cogent Education* .2020;7(1) : 10.1080/2331186X.2019.1690232 10.1080/2331186X.2019.1690232

[4] : Factors influencing study engagement during the COVID-19 pandemic: A cross-sectional study among health and social professions students. *PLoS One* .2021;16(7) : 10.1371/journal.pone.0255191 e0255191 10.1371/journal.pone.0255191 34314450PMC8315536

# Response

**Published:** 2022-05-09

**Authors:** Houman Goudarzi

**Affiliations:** Hokkaido University, Japan

Dear Dr. Forman Erwin Siagian

 Thank you very much for reviewing our manuscript and providing very instructive and scientific comments. We are very thankful and greatly appreciate your comments. As you mentioned, our study has some limitations, and we believe that stating such limitations improves the clarity and quality of scientific papers. We will update the manuscript according to your valuable comments. In the Discussion section, we will state the limitation of the current study not to measure the underlying reasons for declined interest of our medical students in studying/working abroad during the pandemic. As kindly indicated, it is necessary to conduct further quantitative research to clarify the underlying reasons. We are also very thankful for the provided references, which let us discuss these limitations more efficiently. Currently, we are waiting for comments from another reviewer. After receiving comments from all reviewers, we will be back to you with the updated manuscript.

 Thank you very much again for your time, and valuable comments, and for providing new insight for future research projects.   Sincerely,   Houman Goudarzi, M.D., Ph.D. Assistant Professor Center for Medical Education and International Relations, Faculty of Medicine and Graduate School of Medicine, Hokkaido University

# Response

**Published:** 2022-06-14

**Authors:** Houman Goudarzi

**Affiliations:** Hokkaido University, Japan

**Comments from Prof. Forman Erwin Siagian**  

**Firstly, I would like to express my compliment to these authors due to their effort to examine the impact of covid on global medical education and combine it with the plans/futures of Japanese medical students.**

 Response: Thank you very much for accepting the invitation to do a peer review of our manuscript. Your instructive comments helped us improve the quality and clarity of the manuscript, especially in addressing the current study's limitations. We are very thankful to you. We have responded to your comments one by one as follows.    

**This research positively reveals how higher education, especially medicine, has responded to the pandemic for its survival. In my opinion, the limitations of the research have been acknowledged partly, due to probably because of the qualitative approach to writing and this does not become an obstacle for the whole article in contributing positively to the field of medical science. The reason for my statement "the limitations of the research have been acknowledged partly, due to probably due to the qualitative approach to writing" is as follows:  In the conclusion section, the author states that covid reduces the interest of medical students to go study abroad. The underlying reason for this is not stated clearly, I think this is probably because the possible answer is not covered in the questionnaires distributed. So this is a research limitation that has not been revealed in this study yet and further quantitative research is needed**.  

 Response: Thank you very much for indicating this point. As the reviewer stated, the underlying reason (s) for the decline in interest of our students studying abroad during the pandemic was not covered by our questionnaire. Accordingly, we acknowledged this limitation in the Discussion as follows (page 10, first paragraph):

*The current study has some limitations. We collected data from students between 2016 and 2019 via paper-based questionnaires. However, because of the prevalence of remote education in 2020, we collected such information using online questionnaires. This could influence the results; however, based on the adequate orientation of the students before completing the survey and monitoring of the students during the course, comparing the results with the survey conducted at the end of the course suggested a minimal possibility of such bias. In addition, we did not specifically examine the underlying cause(s) for the significant decline in interest in studying or working abroad among the students' in 2020. Therefore further quantitative research is needed to underpin the underlying reasons in detail.*

**I quote Queirós (2017)1, who states "Qualitative research is therefore concerned with aspects of reality that cannot be quantified, focusing on the understanding and explanation of the dynamics of social relations. Qualitative research works with the universe of meanings, motives, aspirations, beliefs, values, and attitudes, which corresponds to a deeper space of relationships, processes, and phenomena that cannot be reduced to the operationalization of variables." Scientific research adopts qualitative and quantitative methodologies in the modeling and analysis of numerous phenomena. The qualitative methodology intends to understand a complex reality and the meaning of actions in a given context. On the other hand, the quantitative methodology seeks to obtain accurate and reliable measurements that allow statistical analysis. Both methodologies offer a set of methods, potentialities, and limitations that must be explored.  In the discussion section, the author states that "It is a very concerning result which could be, at least partially, because of the loss of motivation of the students during the COVID-19 era." It would be very interesting to carry out further research to find out whether it is the loss of motivation that underlies the unwillingness of medical students to learn foreign languages or perhaps any other reason.2 Demotivation has negative effects on language learning processes and outcomes.3 Actually, many factors influence the study engagement of health and social professions students during the COVID-19 pandemic. 4 That's my opinion regarding the limitations of this research, but it should be understood that my personal opinion aims to make this article better. ** 

 Response: Thank you very much for your explanation and for introducing exciting references. I read the report of Queirós et al. (2017) regarding the strength and limitations of qualitative and quantitative research methods with great interest. Also, we appreciate your comments about further studies to explore underlying reasons for unwillingness to learn foreign languages among medical students in future studies. We included your comments on conducting further research on factors influencing demotivation of the students and its consequences in the Discussion, as follows (page 8, first paragraph):  

*Across the five years of the study, around 16% of students who participated in 2020, reported they did not study English, which was the highest compared to the other years ( Figure 2, 16.3% vs. 7.5% in 2019). It is a very concerning result which could be, at least partially, because of the loss of motivation of the students during the COVID-19 era. Further studies are necessary to demonstrate underlying factors that result in the unwillingness of medical students to learn foreign languages, including demotivation (Riasati et al., 2018). Such demotivation due to the pandemic may have consequent language learning failure for medical students (Vakilifard et al., 2020). A recent German study reported that suitable digital learning formats and social support are important study resources for engagement during the COVID-19 pandemic among health and social professions students (Koob et al., 2021). In addition, universities and health institutions should provide beneficial teaching formats and innovative methods to support students during major life events.*

# Peer review: Reviewer response for version 1

**DOI:** 10.21956/mep.20310.r31735

**Published:** 2022-03-28

**Authors:** Jay Yang, MD, PhD, MSEE

**Affiliations:** 1 Emeritus Professor, University of Wisconsin, Madison, WI, USA

Goudarzi *et al. *report on the longitudinal trend of medical students at the Hokkaido University School of Medicine surveyed by questionnaires conducted over 2016 to 2020. The survey focused on three areas of previous English language exposure, the current method of studying English, and plans for international academic experience. The responses captured an abrupt reversal of outward-bound trend in 2020, from that seen during 2016-2019 presumably from the impact of Covid-19. This abatement of interest in international exposure was broad, spanning all categories queried, including the desire to pass an English language skills examination presumably not directly limited by the Covid-related travel restrictions.

 While many research papers have been published on the impact of Covid-19 on medical education, a report focusing on the outward-bound attitude of Japanese medical students is new and well-worth publishing and disseminating the findings.

 Several points for consideration and revision are suggested:

1. As the authors acknowledge in the Discussion, the current result is based on a single questionnaire survey conducted with medical students at an early stage in their medical education. As in most parts of the world, medical education is a 6-year continuum upon graduation from high school. Therefore, the target cohort of “second-year students” is equivalent to a college sophomore in the United States, and attitudes towards future career paths may well change for students at this early stage of education. The current study should be followed up by the collection of data to see whether “intent” translates into “action” by following the post-graduate medical career choices of these cohorts.
2. The current survey is based on a single institution in Japan. While the Hokkaido University School of Medicine is a prominent national university and can be considered as a prototypical School of Medicine in Japan, the curriculum is mostly traditional in contrast to the highly innovative recently opened International University of Health and Welfare at Narita where the first two-year curriculum is taught entirely in English and passing the USMLE is required for graduation. The potential limitation of the current study and whether the attitudes of medical students at Hokkaido University can be extrapolated to all of Japan should be discussed. 
3. The drive towards internationalization of medical education and training in Japan is well-summarized in the Introduction. A key high-impact publication summarizing the need for a transformation of medical education in an interdependent world should be included (Frenk *et al*., Lancet 2010; 376: 1923-58) ^1^. While both are wealthy and economically advanced industrial nations, medical education, training, and indeed the overall medical systems in the United States and Japan are very different, with both systems having advantages and disadvantages. International exposure of young physicians and direct learning in the two disparate systems will surely bring about the integration and the betterment of the medical systems in both countries. However, it is also well known that Japanese MDs face a huge hurdle in successfully matching with US clinical training programs because of cultural and linguistic differences (Heist, J Gen Fam Med, 2020; 21:102-108) ^2^. The traditional Japanese medical establishments may marginalize the repatriated US trained Japanese physicians from implementing meaningful change based on their newly gained knowledge (Heist, Teaching Learning in Medicine 2019; 31:412-423) ^3^. Such systemic limitations could be a more serious threat to the internationalization of Japanese medicine, far outlasting the impact of Covid-19.
4. The tremendous stress on the health care, education, and delivery created by the Covid-19 pandemic is not all bad because it provided an opportunity to reassess the entire healthcare preparedness, delivery, and even the medical education process itself (Lucey, JAMA 2020; 324:1033-1034; ^4^ www.youtube.com/watch?v=qDBtkq42fW8). As with many occasions, the crisis could be a trigger to reassess the status quo and for implementing the long-needed reforms. Such a glimmer of hope in the times of Covid-19 could be mentioned in the discussion.
5. The findings on previous and current English language exposure (Fig 1 and 2) are interesting. How does the result compare with the mode of English language exposure of the non-medical Japanese youths? Is the current finding something unique to the medical professionals?
6. Statistical analysis is properly mentioned in the Methods, but no statistics appear in the proportions data (i.e. All Figs).

Have any limitations of the research been acknowledged?

Partly

Is the work clearly and accurately presented and does it cite the current literature?

Yes

If applicable, is the statistical analysis and its interpretation appropriate?

No

Are all the source data underlying the results available to ensure full reproducibility?

Yes

Is the study design appropriate and is the work technically sound?

Yes

Are the conclusions drawn adequately supported by the results?

Yes

Are sufficient details of methods and analysis provided to allow replication by others?

Yes

Reviewer Expertise:

Cellular and molecular medicine, clinical anesthesiology, medical education

I confirm that I have read this submission and believe that I have an appropriate level of expertise to confirm that it is of an acceptable scientific standard, however I have significant reservations, as outlined above.

## References

[1] : Health professionals for a new century: transforming education to strengthen health systems in an interdependent world. *The Lancet* .2010;376(9756) : 10.1016/S0140-6736(10)61854-5 1923-1958 10.1016/S0140-6736(10)61854-5 21112623

[2] : Japanese International Medical Graduates and entrance into US clinical training: Challenges and methods to overcome them. *J Gen Fam Med* .2020;21(4) : 10.1002/jgf2.332 102-108 10.1002/jgf2.332 32742898PMC7388659

[3] : Contrasting Residency Training in Japan and the United States From Perspectives of Japanese Physicians Trained in Both Systems. *J Grad Med Educ* .2019;11(4 Suppl) : 10.4300/JGME-D-18-01046 125-133 10.4300/JGME-D-18-01046 31428269PMC6697274

[4] : The Transformational Effects of COVID-19 on Medical Education. *JAMA* .2020;324(11) : 10.1001/jama.2020.14136 1033-1034 10.1001/jama.2020.14136 32857137

# Response

**Published:** 2022-04-13

**Authors:** Houman Goudarzi

**Affiliations:** Hokkaido University, Japan

Dear Professor Jay Yang, Thank you very much for your time and instructive comments. We greatly appreciate it. Currently, we are waiting for the comments from other reviewers. After receiving other comments, we will update the manuscript according to your valuable comments. Thank you very much again. Houman Goudarzi

# Response

**Published:** 2022-06-14

**Authors:** Houman Goudarzi

**Affiliations:** Hokkaido University, Japan

**Comments from Prof. Jay Yang**  

**Goudarzi et al. report on the longitudinal trend of medical students at the Hokkaido University School of Medicine surveyed by questionnaires conducted over 2016 to 2020. The survey focused on three areas of previous English language exposure, the current method of studying English, and plans for international academic experience. The responses captured an abrupt reversal of outward-bound trend in 2020, from that seen during 2016-2019 presumably from the impact of Covid-19. This abatement of interest in international exposure was broad, spanning all categories queried, including the desire to pass an English language skills examination presumably not directly limited by the Covid-related travel restrictions. While many research papers have been published on the impact of Covid-19 on medical education, a report focusing on the outward-bound attitude of Japanese medical students is new and well-worth publishing and disseminating the findings.**

 Response: Thank you very much for reviewing our manuscript. We greatly appreciate your instructive comments. We updated the manuscript according to your valuable comments, and we hope you will find it favorable.

**Several points for consideration and revision are suggested: 1) As the authors acknowledge in the Discussion, the current result is based on a single questionnaire survey conducted with medical students at an early stage in their medical education. As in most parts of the world, medical education is a 6-year continuum upon graduation from high school. Therefore, the target cohort of "second-year students" is equivalent to a college sophomore in the United States, and attitudes towards future career paths may well change for students at this early stage of education. The current study should be followed up by the collection of data to see whether "intent" translates into "action" by following the post-graduate medical career choices of these cohorts.**

 Response. As the reviewer mentioned correctly, we also believe that only a single questionnaire survey during preclinical clerkship is not enough to assess the potential effect of the pandemic on future career planning. Therefore, it is necessary to do multi-center studies and follow up with the students longitudinally to observe whether this pandemic affects the "action" of the study participants in the future, not just intention. Therefore, we included this point in the manuscript as a future plan on page 10, first paragraph, as follows:

*We conducted this survey before clinical clerkship, and attitudes toward future career paths may change for the second-grade medical students over time. Therefore, it is necessary to follow up with the students to examine whether intent translates into action after graduation.*

**2) The current survey is based on a single institution in Japan. While the Hokkaido University School of Medicine is a prominent national university and can be considered as a prototypical School of Medicine in Japan, the curriculum is mostly traditional in contrast to the highly innovative recently opened International University of Health and Welfare at Narita where the first two-year curriculum is taught entirely in English and passing the USMLE is required for graduation. The potential limitation of the current study and whether the attitudes of medical students at Hokkaido University can be extrapolated to all of Japan should be discussed.**

 Response: Thank you very much for pointing out this important issue. Our survey in a national university may not be extrapolated to all medical schools in Japan, including the International University of Health and Welfare (Narita campus), with different curriculum and education systems. Therefore, we mentioned this point as a limitation of the manuscript (page 10, first paragraph), as follows:

*Our survey is based on a single medical school in Japan. Therefore, medical students' attitudes in our university may not be extrapolated to all national and private medical schools in Japan. Further multi-institutional surveys are necessary to replicate our results.*

**3) The drive towards internationalization of medical education and training in Japan is well-summarized in the Introduction. A key high-impact publication summarizing the need for a transformation of medical education in an interdependent world should be included (Frenk et al., Lancet 2010; 376: 1923-58)1. While both are wealthy and economically advanced industrial nations, medical education, training, and indeed the overall medical systems in the United States and Japan are very different, with both systems having advantages and disadvantages. International exposure of young physicians and direct learning in the two disparate systems will surely bring about the integration and the betterment of the medical systems in both countries. However, it is also well known that Japanese M.D.s face a huge hurdle in successfully matching with U.S. clinical training programs because of cultural and linguistic differences (Heist, J Gen Fam Med, 2020; 21:102-108)2. The traditional Japanese medical establishments may marginalize the repatriated U.S.-trained Japanese physicians from implementing meaningful change based on their newly gained knowledge (Heist, Teaching Learning in Medicine 2019; 31:412-423)3. Such systemic limitations could be a more serious threat to the internationalization of Japanese medicine, far outlasting the impact of Covid-19**.

 Response: Thank you for introducing the report of Frenk et al. (2010). We included this critical report in the Introduction, as follows (page 3, first paragraph):

*In the 21st century, with extraordinary rapid changes, reforming professional education is inevitable. It is necessary to adapt education to strengthen health systems based on transnational, multi-professional, and long-term perspectives to respond needs of humans (Frenck et al. 2010).*

 Also, thank you very much for indicating report of Heist *et al*. ( *J Gen Fam Med, *2020). We included this in the Introduction, as follows (page 3, second paragraph):

*A recent study examined the U.S. clinical experience for Japanese international medical graduates and reported the benefits of U.S. training (Heist et al., 2020). Although medical education and the overall medical systems in the U.S. and Japan are different, international exposure of young physicians and learning in the two disparate systems will bring about the integration and the improvement of the medical systems in both countries.*

 Regarding the report of Heist et al. ( *Teaching Learning in Medicine*, 2019), there would be several obstacles to the internationalization of Japanese medicine, such as system limitations. However, this is out of the scope of the current manuscript, and discussing this issue may not help this article. Hopefully, we will be able to focus on this issue in the following projects. Again, thank you very much for your precious comments.

**4) The tremendous stress on the health care, education, and delivery created by the Covid-19 pandemic is not all bad because it provided an opportunity to reassess the entire healthcare preparedness, delivery, and even the medical education process itself (Lucey, JAMA 2020; 324:1033-1034;4 www.youtube.com/watch?v=qDBtkq42fW8). As with many occasions, the crisis could be a trigger to reassess the status quo and for implementing the long-needed reforms. Such a glimmer of hope in the times of Covid-19 could be mentioned in the Discussion**.

 Response: As the reviewers stated, this pandemic would be a great opportunity to transform medical education. To emphasize the importance of such an opportunity, we updated the Discussion as follows (page 9, fourth paragraph):

*Although the pandemic stressed the health care systems and medical education, it could also be a catalyst for the transformation of medical education (Lucey et al. 2020). The tremendous stress of the pandemic on education and healthcare systems may provide an opportunity to reassess the entire medical education, healthcare preparedness, and delivery. Moreover, COVID-19 has been pushing universities to change their attitudes toward the curricula, methods of teaching, learning, and assessment. Hopefully, the lessons from the COVID-19 pandemic will force education to a higher level and help the preparedness of universities for pandemics or a global health crisis in the future.  *  

**5) The findings on previous and current English language exposure (Fig 1 and 2) are interesting. How does the result compare with the mode of English language exposure of the non-medical Japanese youths? Is the current finding something unique to the medical professionals?**

 Response: Regarding English language exposure, we did a literature review. However, we did not find any comprehensive study or report regarding English language exposure of Japanese students, including non-medical students. Schools in Japan offer a common (general education-oriented) curriculum for all students from elementary to high school under a single-track system. At the upper secondary education level, students have the option of academic (general), vocational, and specialized programs. Still, the content of the curricula does not vary widely with the type of program, including English education and exposure ways to the English language. However, there is a possibility that some medical students planning to study/work/live abroad have tried more ways to learn English and be exposed to English. Also, medical students who have probably highly educated parents could have more chances to live or visit abroad and learn English more. Further studies would help address any possible difference between medical and non-medical students in Japan regarding previous and current English exposure mode.    

 6) Statistical analysis is properly mentioned in the Methods, but no statistics appear in the proportions data (i.e. All Figs).

 Response: Thank you very much for the comments. We have shown data with percentages and means with standard deviation in Table 1, as indicated in the legend. Also, we showed the descriptive data in percentages for all figures (Fig. 1, 2, and 3).
